# Insight into the Carbon Monoxide Reduction Reaction on Cu(111) from *Operando* Electrochemical X‐ray Photoelectron Spectroscopy

**DOI:** 10.1002/anie.202506402

**Published:** 2025-07-16

**Authors:** Bernadette Davies, Fernando Garcia‐Martinez, Christopher M. Goodwin, David Degerman, Markus Soldemo, Patrick Lömker, Vladimir Grigorev, Sara Boscolo Bibi, Harri Ali‐Löytty, Robin Y. Engel, Joakim Halldin Stenlid, Gabriel L. S. Rodrigues, Tony Hansson, Christoph Schlueter, Xiaodong Zou, Anders Nilsson, Sergey Koroidov

**Affiliations:** ^1^ Department of Chemistry Stockholm University Svante Arrhenius väg 16C, 114 18 Stockholm Sweden; ^2^ Department of Physics Stockholm University AlbaNova University Center Stockholm SE‐106 91 Sweden; ^3^ Photon Science Deutsches Elektronen‐Synchrotron DESY Notkestr. 85 22607 Hamburg Germany; ^4^ Faculty of Engineering and Natural Sciences Tampere University P.O. Box 692 Tampere FI‐33014 Finland; ^5^ Wallenberg Initiative Materials Science for Sustainability Department of Physics Stockholm University Stockholm SE‐114 28 Sweden; ^6^ Department of Chemistry and Chemical Engineering Chalmers University of Technology Kemivägen 10, 412 96 Gothenburg Sweden; ^7^ Department of Physics Chemistry and Pharmacy University of Southern Denmark Campusvej 55, DK–5230 Odense M Denmark; ^8^ Present address: ALBA Synchrotron Light Facility Carrer de la Llum 2, 26, 08290 Cerdanyola del Vallès Barcelona Spain; ^9^ Present address: Liquid Sun Ltd Tekniikankatu 1 Tampere FI‐33720 Finland

**Keywords:** CO reduction, CO_2_ reduction, Electrochemistry, Heterogeneous catalysis, Photoelectron spectroscopy

## Abstract

In this work, we introduce a modified dip‐and‐pull electrochemical X‐ray photoelectron spectroscopy (ECXPS) approach that offers new mechanistic insight into the alkaline carbon monoxide reduction reaction (CORR) over a Cu(111) single crystal surface. We tackle two major unresolved questions in the CORR mechanism that persist in the literature. Firstly, we address the mechanism for methane formation on Cu(111) and show that the mechanism likely proceeds via atomic carbon, which subsequently couples, leading to the accumulation of amorphous carbon on the surface. Secondly, we provide insight into whether the mechanism for acetate formation occurs entirely on the surface or partially within the solution phase, showing that acetate is present on the surface, indicating a surface‐based reaction. These insights into surface‐based mechanisms provide a handle for designing future catalysts that can efficiently target the binding of specific intermediates. Furthermore, we expect that our modified approach to dip‐and‐pull ECXPS – in which we have changed the electrode geometry, the method of introducing the reactant gas and used hard x‐rays – will significantly expand the technique's applicability, enabling studies of the CO_(2)_RR and beyond.

## Graphical abstract

In this work, a modified dip‐and‐pull electrochemical X‐ray photoelectron spectroscopy (XPS) approach is presented and used to probe the alkaline CO reduction reaction (CORR) over a Cu(111) surface. The results provide insight into the methane and acetate formation mechanisms and demonstrate a successful application of the method to the study of the CO_(2)_RR under *operando* conditions.

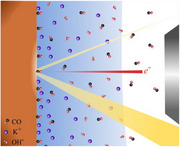

## Introduction

The electrochemical CO_2_ reduction reaction (CO_2_RR) is a promising approach towards making the production of fuels and feedstock chemicals sustainable. If such technologies are to be developed, better understanding is required of the CO_2_RR mechanism. Gaining this understanding, though, presents a significant challenge. The CO_2_RR is a complex process involving many proton and electron transfers, and has more than a dozen potential products accessible via a multitude of possible mechanistic pathways.^[^ ^1^, ^2^ ^]^ The only monometallic catalyst known to produce valuable multi‐carbon and oxygenated products from the CO_2_RR is Cu. The efficiency and selectivity of these catalysts are governed by a complex interplay of effects, and much scientific effort has been directed towards enhancing the selectivity of new Cu‐based catalysts towards valuable C_2+_ products such as ethylene, ethanol and acetate with high Faradaic efficiencies.^[^ ^3^, ^4^, ^5^, ^6^, ^7^, ^8^, ^9^, ^10^ ^]^

It is widely accepted that the first stage in the CO_2_RR towards valuable products is the formation of CO.^[^ ^2^ ^]^ For this reason, the CO reduction reaction (CORR) is often used as a model reaction for the CO_2_RR on Cu‐based materials, allowing a simplified reaction route to be investigated while still providing information that is directly applicable to the direct CO_2_RR. Furthermore, a tandem approach towards CO_2_ reduction, in which CO_2_ is first reduced to CO and then the CO is further reduced to the target products, is increasingly being recognized as a route towards greater overall efficiencies.^[^ ^11^ ^]^ The CORR can be performed under more favourable alkaline conditions^[^ ^12^ ^]^ because it bypasses the need to use aqueous CO_2_, which predominantly forms carbonates under alkaline conditions.^[^ ^11^ ^]^

While ex situ and theory‐based approaches provide an important foundation for hypothesis building, in situ/*operando* measurements are crucial in providing experimental verification of the proposed CORR mechanisms including aspects depending on the operating conditions. Hard X‐ray absorption spectroscopy (XAS) is the most popular technique for such studies^[^ ^13^, ^14^, ^15^ ^]^ because it is readily adaptable to in situ/*operando* measurements. However, traditional XAS provides an averaged signal from the bulk and the surface of the catalyst, making much of the information relating to processes at the electrocatalytic interface challenging to obtain, and is not sensitive towards lighter elements such as carbon and oxygen. Among non‐X‐ray based spectroscopic techniques, surface‐enhanced vibrational spectroscopies are perhaps the state‐of‐the‐art for in situ/*operando* studies of catalytic interfaces during the CO_2_RR/CORR.^[^ ^16^, ^17^, ^18^, ^19^ ^]^ However, these can only probe species that have Raman and/or IR active vibrational modes with relatively high cross‐sections. X‐ray photoelectron spectroscopy (XPS) is a particularly powerful tool for characterizing catalytic interfaces because it allows the state of the catalyst and almost all chemical species to be tracked simultaneously and is inherently surface specific. In the last decade, XPS has been adapted for the study of electrochemical interfaces.^[^ ^20^, ^21^, ^22^, ^23^, ^24^, ^25^ ^]^

One popular approach to electrochemical XPS (ECXPS) measurements is the *dip‐and‐pull* method; the electrode material of interest is “dipped” into a reservoir of electrolyte and “pulled” partially out to produce a thin film on the surface through which the electrode‐electrolyte interface can be measured.^[^ ^21^ ^]^ This approach is relatively straightforward (compared to, for example, the suspended droplet approach)^[^ ^26^ ^]^ and allows direct probing of the relevant interface (as opposed to thin‐electrode cell‐based approaches).^[^ ^27^ ^]^ Despite some notable successes in using dip‐and‐pull ECXPS to study electrocatalytic reactions,^[^ ^28^, ^29^, ^30^, ^31^ ^]^ this technique has yet to be explored for its potential to provide insight into the CORR or CO_2_RR mechanism. The scope of dip‐and‐pull ECXPS studies has been limited by technical challenges, primarily related to mass transport limitations through thin electrolyte films.

**Figure 1:**
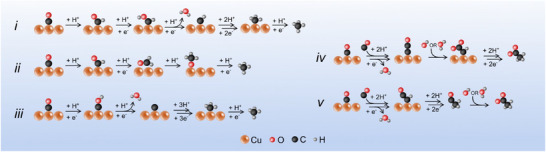
Schematic of some simplified prosed mechanisms for (i–iii) methane formation and (iv,v) acetate formation presented in the literature. i) The CHO pathway for methane formation,^[^ ^8^, ^34^, ^35^, ^36^ ^]^ ii) the CHO pathway for methane formation that proceeds via formaldehyde,^[^ ^37^, ^38^ ^]^ iii) the COH pathway for methane formation that proceeds via atomic carbon on the surface,^[^ ^39^, ^40^ ^]^ iv) the formation of acetate via a ketene intermediate that undergoes a surface‐based carboxylation,^[^ ^41^ ^]^ v) the formation of acetate via a ketene intermediate that desorbs from the surface and undergoes carboxylation in the bulk electrolyte.^[^ ^42^, ^43^, ^44^ ^]^

The Cu(111) surface is an important model catalyst for the CORR and has been widely studied to understand the structure‐activity relationship of the reaction mechanism. It is well‐established that the atomically flat Cu(111) facet produces mostly methane, while more open facets produce higher proportions of ethylene and ethanol.^[^ ^32^, ^33^ ^]^ Despite this well‐known facet‐selectivity, there remains a lack of consensus on the mechanism for methane formation on Cu(111). Some studies propose that it proceeds via the formation of a ‐CHO intermediate, that is then either reduced to a ‐CH intermediate via ‐CHOH (Figure 1i),^[^ ^8^, ^34^, ^35^, ^36^ ^]^ or via the formation of a CH_2_O (formaldehyde) intermediate that is subsequently reduced to an ‐OCH_3_ intermediate (Figure 1ii),^[^ ^37^, ^38^ ^]^ while others posit that it proceeds via a ‐COH intermediate which is reduced to atomic carbon (Figure 1iii).^[^ ^39^, ^40^ ^]^

Acetate was, until relatively recently, considered to only be a minor product of the CORR/CO_2_RR.^[^ ^12^ ^]^ However, it has been recently shown that the CORR can be tailored towards the selective production of acetate under conditions of high pH,^[^ ^42^, ^45^ ^]^ and that the Cu(111) facet is selective towards acetate production under such conditions.^[^ ^43^ ^]^ Because of this finding, the mechanism of acetate formation from the CORR on Cu(111) has started to be explored. One major inconsistency in the existing literature is whether the carboxylate‐forming step in the formation of acetate – proposed to be variously the reaction of a ketene intermediate with H_2_O^[^ ^43^ ^]^ or OH^−[^ ^41^, ^42^, ^44^ ^]^ – occurs on the surface (Figure 1iv)^[^ ^41^ ^]^ or in the bulk electrolyte (Figure 1v).^[^ ^42^, ^43^, ^44^ ^]^

In this work, we address these unresolved questions, using a further developed dip‐and‐pull ECXPS method to study the alkaline CORR on Cu(111) *operando*. Our results indicate that methane formation proceeds at least in part via the formation of atomic surface carbon. Furthermore, the spectra suggest that one of the pivotal steps in the mechanism for acetate formation – the formation of the carboxylate group – likely proceeds on the surface, and not in the bulk electrolyte. The modified dip‐and‐pull ECXPS set‐up presented here overcomes the practical limitations that have hindered previous dip‐and‐pull ECXPS studies of catalytic processes: with this study, we demonstrate the potential of this technique for examining the CORR/CO_2_RR on Cu surfaces.

## Results and Discussion

**Figure 2:**
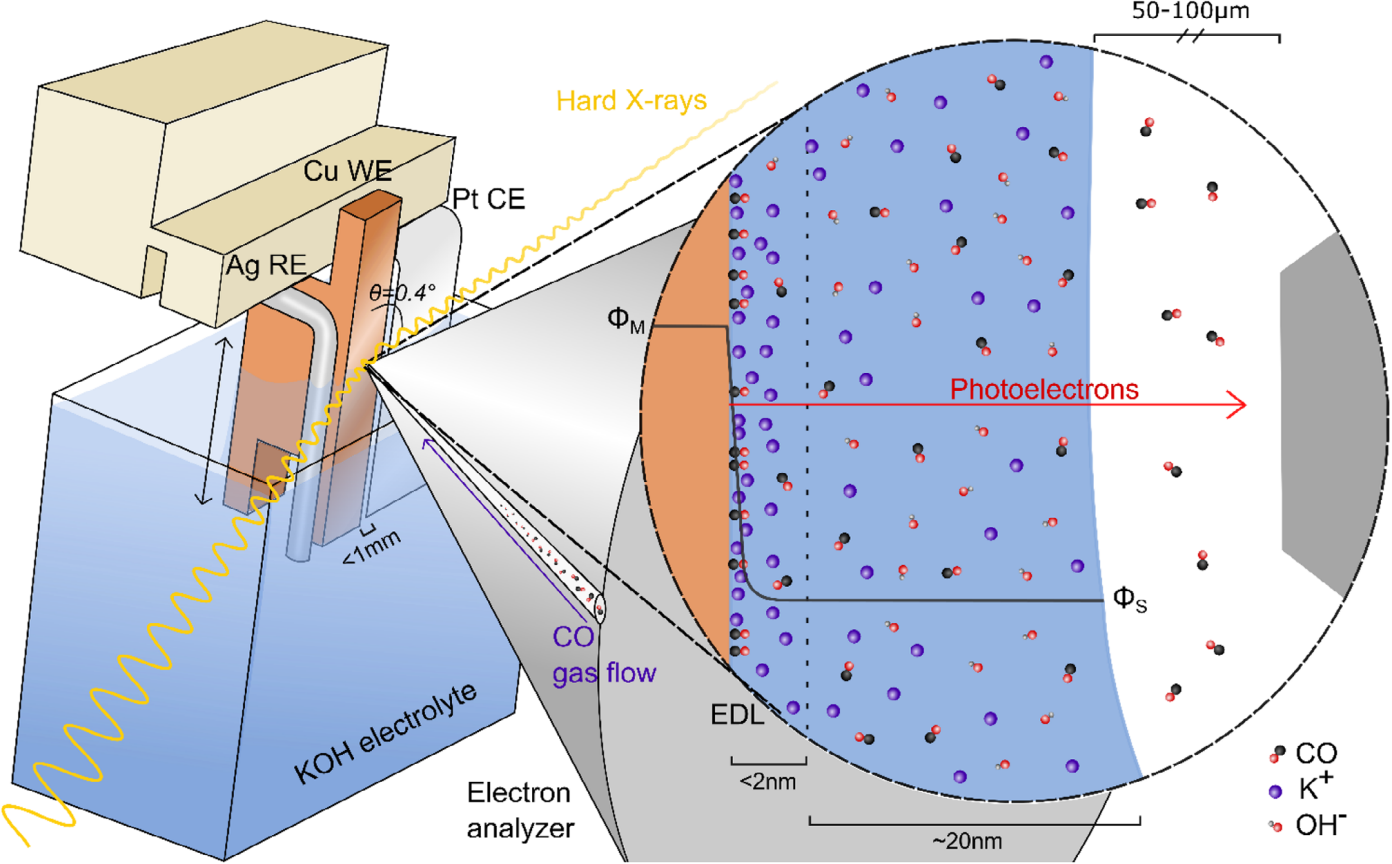
Schematic of the dip‐and‐pull set‐up commissioned within the POLARIS instrument at beamline P22/Petra III, DESY. The three electrodes are held in‐line on the sample holder with a <1 mm gap between to minimize the Ohmic drop. Hard X‐rays with a photon energy of 5.5 keV were used in a grazing incidence geometry (0.4° incidence). CO gas was flown directly towards the measurement position using an aperture adjacent to the nozzle of the electron analyser. The Cu WE is held to the ground, and the potential drop across the electric double layer (EDL) [between the potentials of the metal (ΦM) and the solution (ΦS)] results in a shift of the binding energies of the liquid phase species.

In a dip‐and‐pull ECXPS experiment, the solid‐liquid interface of the working electrode (WE) is probed through a thin meniscus of electrolyte on the surface. The electrochemical environment within the XPS chamber is illustrated in Figure 2. The inelastic mean free path (IMFP) of photoelectrons through water is much shorter than through a scattering gas phase at the same pressure. The maximum thickness of the electrolyte layer for photoelectrons to go through is therefore determined by the energy of the incident X‐rays. If soft X‐rays (< ∼2 keV) are used, the meniscus must be below 10 nm to retain at least 20% of the signal, assuming an exponential attenuation of the signal transmission in accordance with the Beer‐Lambert law (Supplementary Note 1). This experimental restriction of the meniscus thickness can result in severely limited mass transport. For this study, the hard X‐ray (> ∼2 keV) photoelectron spectroscopy (HAXPES) instrument POLARIS at the German electron synchrotron (DESY) is used with a photon energy of 5.5 keV. The IMFP of photoelectrons through water, generated by X‐rays with a photon energy of 5.5 keV, will be approximately 11 nm.^[^ ^46^ ^]^ This means that the thickness of the meniscus in the region probed by X‐rays can be thicker, allowing for a lesser degree of mass transport inhibition while still retaining a viable signal. In this work, meniscuses of up to 21 nm were used (Figure S1 and Supplementary Note 1). For a more detailed discussion of the impact of electrolyte meniscus thicknesses on XPS signal detection, see Supplementary Note 2.

Within a nanometric layer of electrolyte, mass transport is restricted parallel to the interface.^[^ ^47^ ^]^ The long distance between the bulk electrolyte and the measurement position in a typical dip‐and‐pull set‐up can lead to a significant Ohmic drop when measuring at higher Faradaic current densities.^[^ ^31^, ^48^ ^]^ In our electrochemical set‐up, the Cu single crystal WE is held in‐line with the auxiliary electrodes with a <1 mm gap (Figure 2), in order to produce a common meniscus between the electrodes (shown in Figure S2). This minimizes the distance that charge carriers must travel between the bulk electrolyte and the measurement position and thereby limits the Ohmic drop.

The issue of slow mass transport between the bulk electrolyte and nanometric film has restricted the scope of previous dip‐and‐pull studies of electrocatalytic reactions to processes where the primary reactant is water (such as the oxygen evolution reaction) because the rate of consumption of a less abundant reactant typically exceeds the rate at which it can be replenished at the interface.^[^ ^27^ ^]^ To overcome this issue, CO gas was flown directly towards the measurement position using a tube adjacent to the front cone of the electron analyser, see Figure 2. This method of delivery, as opposed to bubbling the CO into the electrolyte, means that CO must only diffuse through the thin electrolyte film perpendicular to the measurement position, where mass transport is fast.

Our inline electrode configuration would be difficult to achieve with a conventional reference electrode such as Ag/AgCl. Instead, an Ag wire pseudo‐reference electrode is used. The stability of the Ag pseudo‐reference was tested by performing successive cyclic voltammograms for several hours to ensure no drift of the potential scale (Figure S3). A Pt counter electrode was used for its flexibility and easy application to our sample holder (Figure S2). Survey spectra recorded after a full set of measurements did not indicate the presence of Pt contamination on the WE (Figure S4). The Cu samples were pre‐treated by electropolishing prior to the ECXPS measurements, according to the procedure outlined by Hori et al.^[^ ^49^ ^]^ This procedure is standard in the literature for studies of Cu single crystal surfaces for the CORR. Electropolishing produces a defect‐rich surface with specific morphological features, resulting in the exposure of more high‐index Cu sites that have been shown to promote both CO binding and CORR activity.^[^ ^7^ ^]^ Prior to any XPS measurements, the Cu samples were reduced using chronopotentiometry in situ, to remove any ambient Cu oxides.

**Figure 3:**
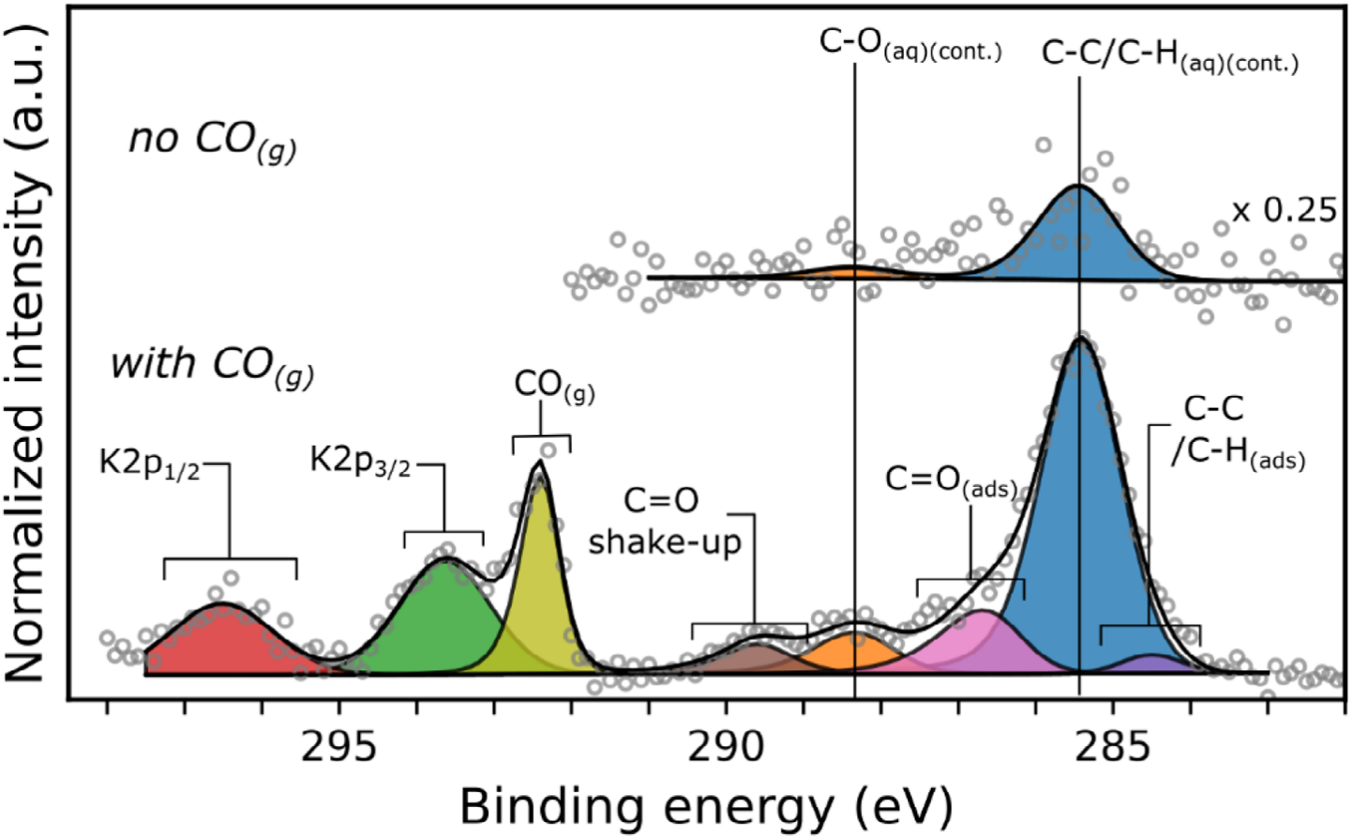
C1s X‐ray photoelectron spectra of the Cu(111) single crystal surfaces at the OCP (+0.13 V vs. RHE) in 0.1 M KOH prior (top) and after (bottom) introducing CO gas flow. The binding energy scale is corrected with respect to the Fermi level of the Cu WE.

CO gas was introduced to the system at the open circuit potential (OCP), measured as + 0.13 V versus the reversible hydrogen electrode (RHE). CO was flown continuously for the duration of the measurements and the chamber pressure maintained at 20 mbar of CO + 20 mbar of H_2_O originating from the vapour pressure of the aqueous electrolyte. Figure 3 shows the C1s X‐ray photoelectron spectra of the Cu(111) surface prior to and while flowing CO gas. There exists some degree of carbon‐based contamination prior to introducing the CO. The hydrocarbon component of this signal (C─C/C─H_(aq)(cont.)_) appears at 285.5 eV at the OCP, and the oxygenated component (C─O_(aq)(cont.)_) at 288.4 eV. Such contamination is common in XPS measurements, especially where H_2_O is used, as has been noted widely in the literature.^[^ ^50^ ^]^ This contamination signal shifts with the applied potential (see later discussion, Figure S5 and Supplementary Note 3). Furthermore, its magnitude scales with meniscus thickness (Figure S6). Together, these observations imply that the carbon contamination is predominantly located within the liquid phase, that is, within the bulk electrolyte and at the liquid‐gas interface. The binding energies of these peaks are consistent with the binding energies of aqueous hydrocarbons as measured in liquid jet XPS studies.^[^ ^51^ ^]^

On introducing CO, a peak emerges corresponding to gas phase CO at 292.4 eV.^[^ ^52^ ^]^ Chemisorbed CO (C═O_(ads)_) produces a main peak at 286.6 eV and a large satellite peak at 289.6 eV.^[^ ^53^, ^54^ ^]^ The final satellite that should appear at 293.7 eV is concealed by larger peaks in the higher binding energy region. These satellites result from *shake‐up* transitions, where a portion of the kinetic energy of the emitted photoelectron is lost to transitions of the valence electrons within the molecular structure.^[^ ^55^ ^]^ This shake‐up structure results in a broadened and asymmetric line‐shape. Aqueous CO has not yet been resolved with XPS and may contribute to the intensity of the peaks within the higher binding energy (> 286.6 eV) region. This contribution is expected to be minor: based on calculated CO coverages (see later discussion), meniscus thicknesses and the expected solution concentration of CO, the aqueous CO signal should have no more than ∼30% of the signal intensity of the surface‐phase CO signal (see Supplementary Note 4 and Figure S7). Peaks at 293.6 and 296.5 eV correspond to the K 2p_3/2_ and K 2p_1/2_ signals from the K^+^ on the surface and in the electrolyte.^[^ ^56^ ^]^

Interestingly, an additional component appears at 284.5 eV, assigned to surface phase amorphous carbon (C─C/C─H_(ads)_).^[^ ^53^, ^57^, ^58^ ^]^ The emergence of this feature has been observed by other studies under non‐electrochemical conditions, and appears as a result of low barrier CO dissociation, which produces atomic carbon.^[^ ^59^, ^60^ ^]^ This atomic carbon couples rapidly, producing the amorphous carbon signal. On stepped Cu(211) surfaces, the CO dissociation has been attributed to CO preferentially binding on C atoms that exist on undercoordinated sites, after which it is able to undergo a low barrier Boudouard reaction (2CO_(ads)_ → C_(ads)_ + CO_2_).^[^ ^59^ ^]^ On Cu(100) surfaces, CO adsorption‐induced surface roughening has been proposed to produce a surface that is catalytically active for “direct” CO dissociation (CO → C_(ads)_ + O_(ads)_).^[^ ^60^ ^]^ While unreconstructed Cu(111) would be expected to be inactive for these CO dissociation processes, the roughening of the Cu(111) surface induced by the electropolishing pre‐treatment and/or CO adsorption likely produces undercoordinated Cu sites on the surface that are active for CO dissociation.

The product distribution from the CORR is known to be coverage dependent. Carbon‐carbon coupling processes of chemisorbed CO or products thereof, widely considered to be the pivotal step in the formation of C_2+_ products, are likely to be supported by a close‐packed arrangement of CO molecules.^[^ ^61^, ^62^, ^63^, ^64^, ^65^ ^]^ Conversely, some studies have indicated that conditions of low CO coverage promote the formation of methane.^[^ ^63^ ^]^ The coverage of CO is partially dependent on the partial pressure of CO exposed to the electrolyte. In this study, under a CO partial pressure of ∼20 mbar, we might expect a lower CO coverage on the surface than under the pressure conditions that the CORR would typically be performed, with one atmosphere of CO or higher. It is therefore possible that the formation of ethylene, ethanol and acetate will be reduced because of the experimental conditions (see Discussion below).

On a pristine Cu surface, CO chemisorbs weakly, and desorbs readily well below room temperature.^[^ ^64^ ^]^ In our system, however, the K^+^ ions from the electrolyte will promote CO binding to the surface; alkali metals on the surface have been seen to significantly raise the temperature at which CO is seen to desorb from Cu.^[^ ^65^ ^]^ Recent studies have also demonstrated that CO adsorption on Cu is a potential dependent process, with mild reducing potentials significantly enhancing the coverage of CO.^[^ ^66^, ^67^ ^]^ Furthermore, the reconstruction of the surface due to the pre‐treatment, CO adsorption and applied potential can produce preferential binding sites for CO and so influence the coverage. The exact coverage of CO will depend on the chemical state and structure of the Cu surface, the local pH, the electrolyte and the potential drop at the electrode surface.^[^ ^64^, ^67^, ^68^ ^]^ Coverage calculations indicate a CO coverage of 0.19 ML at the OCP (see Supplementary Note 5).

**Figure 4:**
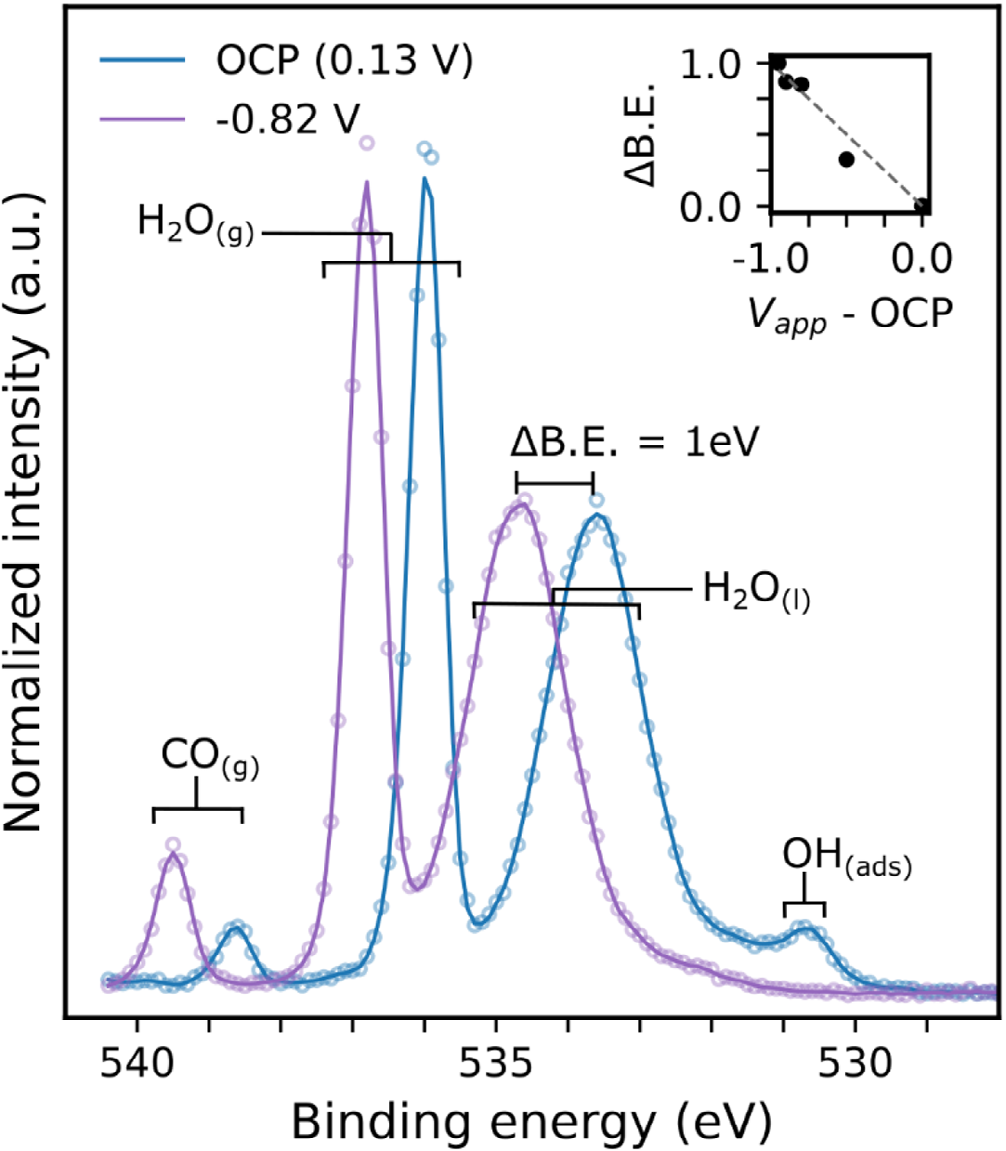
Evolution of the O1s XPS spectrum of the Cu(111) single crystal surface with applied potential, from the OCP (blue) to −0.82 V (purple). An additional ∼0.95 V of applied potential relative to the OCP results in an ∼1 eV shift in the binding energy of the H_2_O(l) peak). The inset shows the relationship between the applied potential (V_app_‐OCP) and the resulting shift in the binding energy (ΔB.E.) of the H_2_O(l) peak for the full range of potentials used in this study (black filled circles) and the expected 1:‐1 relationship (grey dashed line). The spectra are normalized to the intensity of the liquid phase water peak. All potentials are reported versus RHE. The hollow circles show the raw spectral data, the solid lines show the spectral data smoothed by a third order Savitzky‐Golay filter with a window of seven.

Next, we explore the evolution of species at the electrode–electrolyte interface under applied potential. Within the electrochemical cell configuration (Figure 2), the potential is applied with the WE held to the same ground as the electron analyser. Since the Fermi level of the Cu electrode remains constant, the electric field induces a shift in the binding energies of the peaks associated with species present in the electrolyte.^[^ ^23^, ^69^ ^]^ If the species chemisorbed to the surface, situated within the electric double layer (EDL) (Figure 2, inset), are screened by electron transfer from the grounded substrate during ionization, they will remain unaffected by the applied potential (provided there is no chemical change of the corresponding species). Conversely, if chemical species are not chemisorbed with the WE, their peaks will shift according to their location within the EDL.^[^ ^70^ ^]^ A 1:‐1 shift in the binding energies of the bulk liquid‐phase peaks in the XPS spectrum in response to the applied potential is interpreted as evidence of “potential control” at the point of x‐rays impinging on the surface. The evolution of the O1s photoelectron spectrum of the Cu(111) electrode–electrolyte interface with applied potential is shown in Figure 4. The peak centred at 533.7 eV at the OCP arises from liquid phase H_2_O (H_2_O_(l)_). This peak shifts according to the applied potential, demonstrating full potential control even at −0.82 V versus RHE, the strongest reducing potential relevant for this study. This result demonstrates that we have successfully avoided the impact of mass transport limitations of charge carrying species – and the associated Ohmic drop at higher Faradaic current densities – on our results.

**Figure 5:**
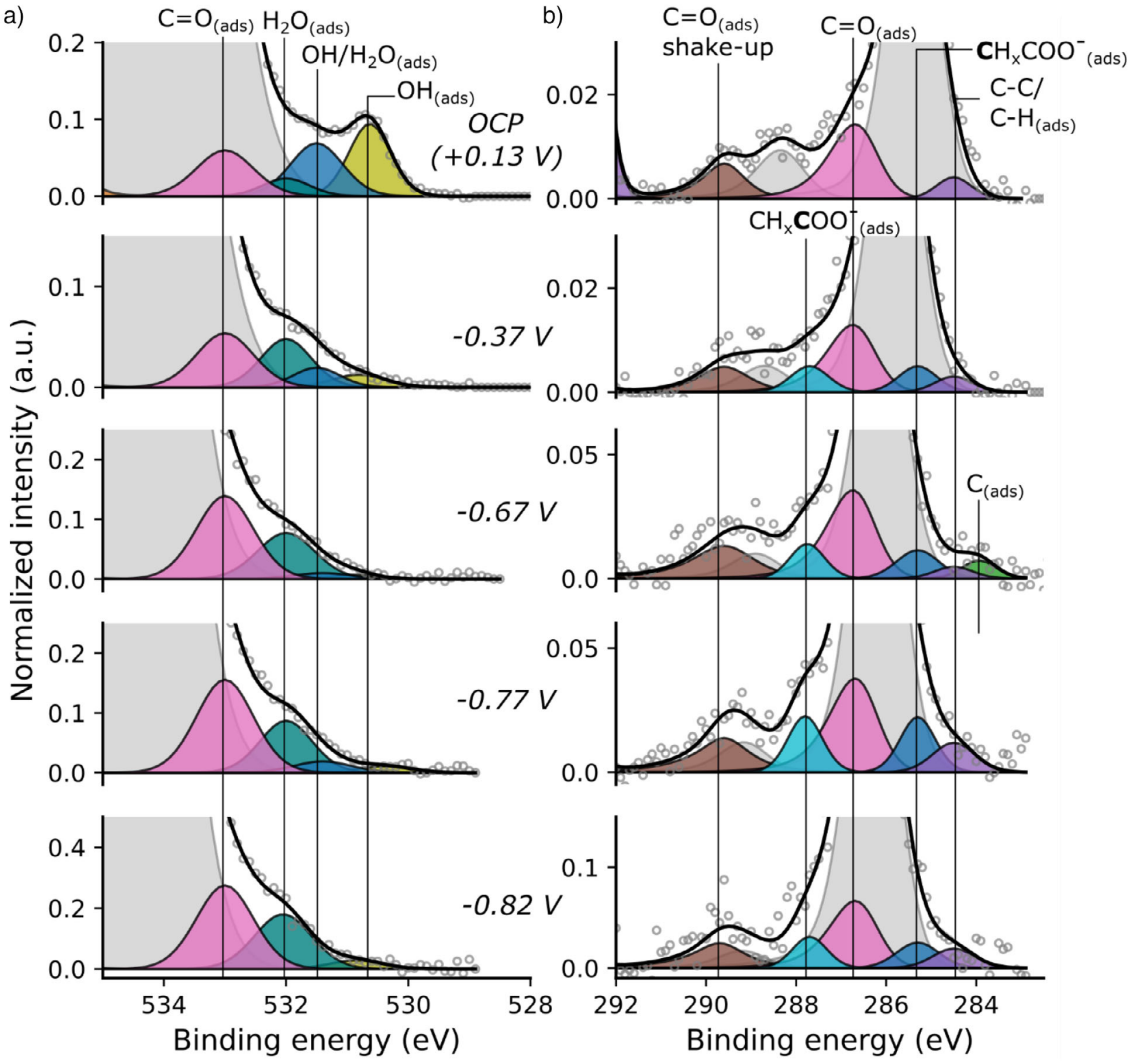
a) O1s and b) C1s XPS spectra recorded on Cu(111) in CO‐saturated 0.1 M KOH as a function of applied potential. The liquid phase peaks are shown in light grey. All potentials are quoted versus RHE. All spectra are normalized to the area of the corresponding Cu2p_3/2_ peak. Note the differences in scaling of the intensity axes. The binding energy scale is with respect to the Fermi level of the Cu WE. Shirely backgrounds have been subtracted.

Figure 5 shows the evolution of the O1s and C1s XP spectra of Cu(111) at a series of successively applied potentials between the OCP (+0.13 V) and −0.82 V versus RHE. At each potential, the spectral regions were scanned continuously while the potential was held for a period of 15–20 min, and the resulting scans averaged. To make the evolution of the relevant surface‐based species clear, the surface components are highlighted in colour while the liquid phase signals are shown in light grey. The full spectra, including the liquid phase signals, are shown in Figure S5 and discussed in Supplementary Note 3. The surface‐phase contributions and liquid‐phase contributions to the spectra can be effectively distinguished based on their behaviour under applied potential, as is discussed above. Between −0.4 and −0.8 V versus RHE is the expected region of productive CORR.^[^ ^12^ ^]^ Figure S8 shows the reduction currents at these potentials and a current‐potential (IV) curve recorded in situ. Vigorous gas evolution on the surface of the electrode impedes the XPS measurements, and therefore, it was not possible to measure the surface at more negative potentials, where the hydrogen evolution reaction begins to compete more efficiently with the CORR.^[^ ^12^ ^]^ The corresponding Cu2p spectra are shown in Figure S9 and demonstrate no chemical change in the Cu WE over the course of the experiments. It should be noted that that the Cu(111) surface may undergo some degree of transformation – for example, surface reconstruction^[^ ^71^, ^72^ ^]^ or the formation of specific morphological features^[^ ^73^ ^]^ – during the CORR process. At present, it is not possible to combine ECXPS with simultaneous structural characterization to allow explicit identification of the active sites for the processes we explore herein.

In the surface region of the O1s spectrum (Figure 5a), peaks at 530.8  and 531.5 eV at the OCP are assigned to surface hydroxide (OH_(ads)_) and surface hydroxide in a hydrogen bonding structure with water (OH/H_2_O_(ads)_), respectively.^[^ ^74^ ^]^ These features are reduced at −0.37 V, but very small signals from both species persist as low as −0.82 V. There is no evidence of surface oxide (Cu─O_(ads)_) species – which would appear at approximately 529.5 eV^[^ ^74^ ^]^ – at any potentials, in contrast to other studies that have indicated the presence of Cu─O_(ads)_ species on polycrystalline Cu under alkaline CORR conditions as low as −0.8 V.^[^ ^17^ ^]^ Other surface phase components at 532  and 533 eV are assigned to adsorbed water (H_2_O_(ads)_)^[^ ^75^ ^]^ and adsorbed CO (C═O_(ads)_),^[^ ^53^ ^]^ respectively. For comparison to the O1s region prior to flowing CO gas, we refer to Figure S10 in the Supporting Information.

**Figure 6:**
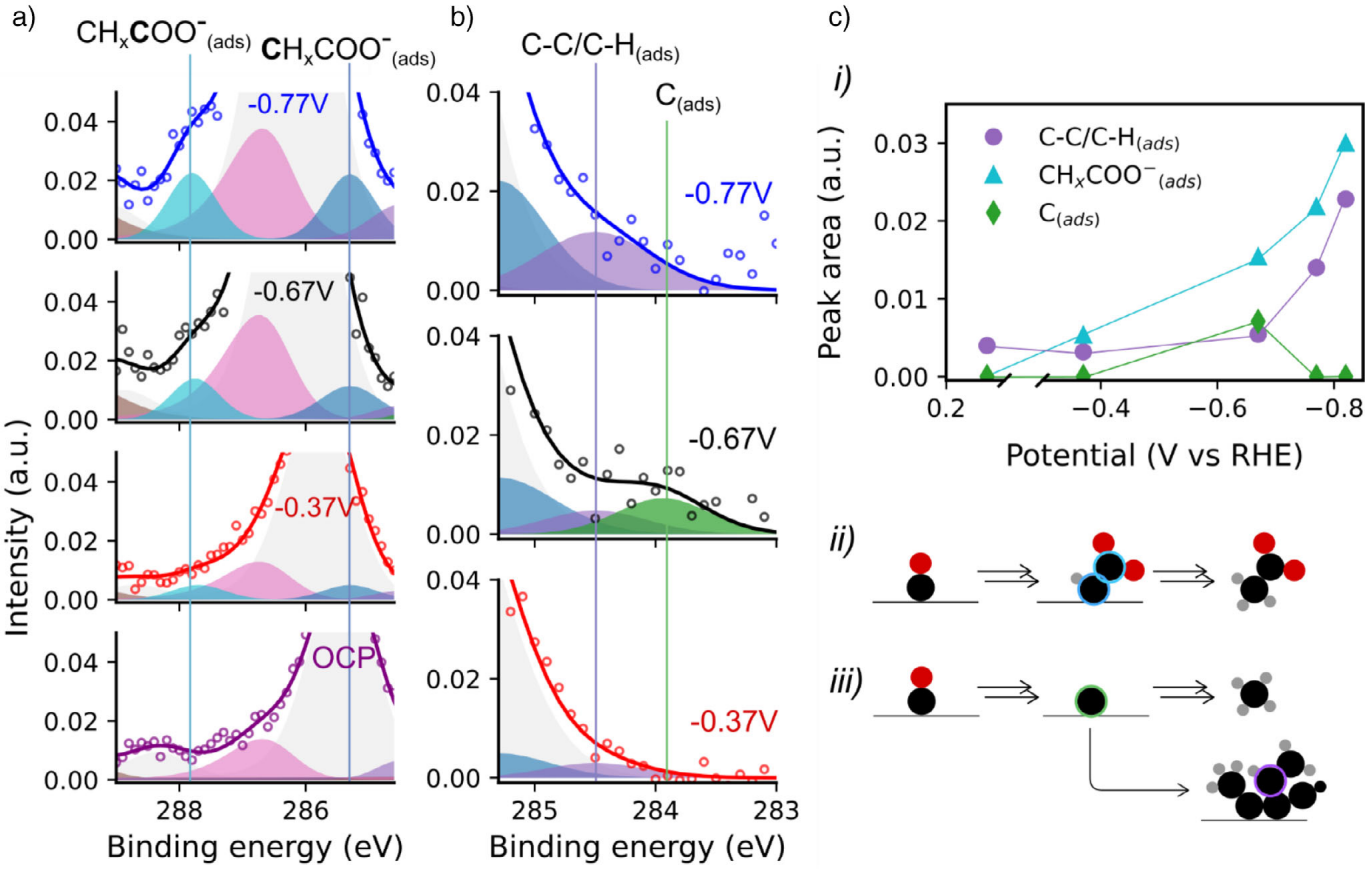
a) The C1s spectra of the Cu(111) surface in the potential range of the OCP to −0.77 V versus RHE, with a narrower binding energy to emphasize the emergence and growth of the acetate (CH_x_COO^−^_(ads)_) peaks. b) The C1s spectra in the potential range of −0.37  to −0.77 V versus RHE, with a narrower binding energy range the emphasize the emergence and disappearance of the atomic carbon peak (C_(ads)_) and the subsequent growth in the amorphous carbon peak. c i) Cu2p normalized peak areas of the amorphous carbon (C─C/C─H_(ads)_), acetate (CH_x_COO^−^_(ads)_) and atomic carbon (C_(ads)_) peaks with applied potential, ii) schematic illustrating the formation of chemisorbed acetate, iii) schematic illustrating the formation of atomic carbon that can form methane or couple, forming amorphous carbon.

In the C1s region (Figure 5b), at −0.37 V, new components are observed at 287.8  and 285.3 eV and are assigned to the carboxylate group (CH_x_ **C** OO^−^_(ads)_) and the CH/CH_2_ group (**C** H_x_COO^−^_(ads)_) in surface phase acetate, respectively. The expected onset potential for acetate formation from the literature lies between −0.2 and −0.4 V versus RHE.^[^ ^42^ ^]^ Figure 6a shows a narrower binding energy region of the spectra on the same intensity scale to more clearly emphasize the emergence and growth of these features. Acetate has yet to be observed on a Cu(111) surface with XPS. Table S1 shows a range of C1s binding energies of acetate species calculated with DFT, and collated values from the literature from either similar species or from acetate on different surfaces, for comparison. In the O1s region, the carboxylate group would be expected to produce a signal in the same binding energy region as the strong H_2_O_(l)_ peak (Figure 5a), and thus is not distinguishable. Both peaks continue to grow significantly at more negative potentials (Figures 5b and 6a,c).

Multiple studies have proposed,^[^ ^41^, ^42^, ^43^, ^45^ ^]^ based primarily on computational studies, that acetate formation under alkaline conditions proceeds via a ketene (CH_x_CO) surface intermediate (Figure 1). This ketene intermediate has been identified on Cu(100) surfaces during the CO_2_RR in alkaline conditions using in situ Raman spectroscopy,^[^ ^16^ ^]^ but within our results would overlap with the higher energy tail of the chemisorbed CO main peak. The surface ketene has then been proposed to react with either OH^−[^ ^41^, ^42^, ^44^ ^]^ or H_2_O^[^ ^43^ ^]^ to form acetate. There has been no consensus, however, on whether this reaction occurs on surface sites (Figure 1iv),^[^ ^41^ ^]^ or whether the ketene intermediate first desorbs and reacts in the bulk electrolyte (Figure 1v).^[^ ^42^, ^43^, ^44^ ^]^ The presence of acetate on the surface in our results demonstrates that the mechanism for acetate formation on Cu(111) must proceed, at least in part, via a wholly surface‐based reaction. It is unlikely that a negatively charged acetate species, once formed in the electrolyte, would migrate and adsorb on to the Cu(111) WE due to electrostatic repulsion. Our observations are therefore consistent with a mechanism for acetate formation in alkaline electrolytes that proceeds predominantly via the reaction of (either solution or surface bound) OH^−^ or H_2_O, with a surface bound ketene intermediate.

At −0.67 V, an additional peak appears at 283.8 eV and is assigned to atomic carbon (C_(ads)_) on the surface (Figures 5b and 6b). This signal does not persist at more negative potentials (−0.77  and −0.82 V), whereupon the amorphous carbon signal at 284.5 eV begins to grow significantly (Figures 5b and  6b,c). A likely explanation for this behaviour is that, at −0.67 V, methane formation begins, and the atomic surface carbon (C_(ads)_) forms as an intermediate along this mechanistic pathway. The onset of methane production from product quantification studies in the literature is approximately −0.65 V.^[^ ^8^ ^]^ At more negative potentials, the formation of atomic carbon in the methanation pathway continues with an increased rate. The accumulation of the amorphous carbon signal can be attributed to the resultant coupling of this atomic carbon when its surface coverage is increased.

This observation provides direct experimental evidence that methane formation, at least partly, proceeds via atomic carbon during CORR activity on Cu(111).^[^ ^39^, ^76^ ^]^ This route was originally proposed to proceed via the electrochemical reduction of CO to C_(ads)_ and OH_(ads)_,^[^ ^40^ ^]^ while more recent studies have proposed that the CO is first reduced to a COH intermediate, which is then further reduced to C_(ads)_, with the OH leaving as H_2_O (Figure 1iii).^[^ ^39^, ^77^, ^78^ ^]^ This mechanism is as opposed to the other major hypothesis, in which there is a non‐electrochemical protonation of the C atom in CO_(ads)_, resulting in the formation of a CHO (formyl) group, which would then be reduced either to CHOH (Figure 1i)^[^ ^8^, ^34^, ^35^, ^36^ ^]^ or CH_2_O (Figure 1ii).^[^ ^37^, ^38^ ^]^ The accumulation of the amorphous carbon signal likely results from the formation of atomic carbon because computational studies have shown that the breaking of C─H bonds – as would be required for continuous C─C coupling of these species – has a high energetic barrier.^[^ ^39^, ^79^ ^]^ Such carbon accumulation from methane formation is known to result in the eventual poisoning of the Cu surface, and has recently been observed using ex situ XPS on polycrystalline Cu surfaces, where it produced a spectral signature very similar to that of carbon black, a mixture of amorphous, mostly combusted hydrocarbons.^[^ ^80^ ^]^

## Conclusion

To conclude, we have presented a modified dip‐and‐pull ECXPS set‐up and demonstrated its capabilities by probing the surface chemistry of the CORR over a Cu(111) single crystal model catalyst in alkaline conditions. The modified approach overcomes the mass transport limitations that have hampered previous studies by using hard X‐rays, minimizing the distances between the electrodes, and supplying the reactant CO gas directly towards the probed interface. We found that the adsorption of CO produced an estimated coverage of 0.19 ML at the OCP, and there was evidence of CO dissociation even without applied bias. This finding has important implications for the CORR process, as it is likely that this process could both contribute to the formation of other CORR products, and to the eventual poisoning of the Cu surface. The results allow us to address two major open questions about the CORR mechanism. Firstly, the results are consistent with a mechanism of methane formation [the major product on Cu(111)] that proceeds via atomic carbon, evidenced by a signal corresponding to atomic carbon on the surface observed at −0.67 V, and the rapid growth of the amorphous carbon signal thereafter, corresponding to the coupling of the atomic carbon that has been observed to poison the surfaces of Cu electrodes. Secondly, the emergence of a peak corresponding to chemisorbed acetate is direct experimental evidence that acetate formation on Cu(111) proceeds at least in part via a mechanistic pathway whereby the carboxylate formation step in the reaction occurs with a surface‐bound, not a solution phase, ketene intermediate.

We expect the developments to significantly expand the scope of dip‐and‐pull ECXPS studies of catalytic reactions, both with regards to the CO_(2)_RR – this study will be extended to other Cu facets and oxidised Cu – and beyond. One of the most challenging aspects of this technique is the overlap of adsorbate peaks with the dissolved contaminations and the signal from the solvent, in the ∼285–286 eV C1s region and the ∼533–536 eV O1s region. Future work should focus on better methods of removing the carbon contamination, to allow better resolution of the full range of the surface‐based carbon‐containing intermediates. Furthermore, it would be worthwhile to explore the impact of reactant gas pressure on these measurements, since the gap between the pressures under which ECXPS can be performed and the pressures under which CO/CO_2_ electrolysers operate could impact the conclusions. This “pressure gap” in ECXPS studies has yet to be explored.

## Supporting Information

The authors have cited additional references within the supporting information.^[^ ^81^, ^82^, ^83^, ^84^, ^85^, ^86^, ^87^, ^88^, ^89^, ^90^, ^91^, ^92^, ^93^, ^94^, ^95^, ^96^ ^]^

## Author Contributions

Conceptualization—B.D., C.M.G., A.N., S.K. Formal analysis—B.D., D.D. Investigation—B.D., F.G.M., C.M.G., D.D., M.S., P.L., V.G., S.B.B., H.A., R.Y.E., C.S., S.K. DFT calculations—G.L.S.R., J.H.S. Project administration—B.D., F.G.M., C.M.G., S.K. Visualization—B.D. Writing—original draft—B.D. Supervision—X.Z., A.N., S.K. Writing—review and editing—all authors.

## Conflict of Interests

The authors declare no conflict of interest.

## Supporting information



Supporting Information

## Data Availability

The data that support the findings of this study are available in the Supporting Information of this article.
